# Associations of modifiable factors with risk of irritable bowel syndrome

**DOI:** 10.3389/fnut.2024.1362615

**Published:** 2024-07-01

**Authors:** Ying Chen, Hong Yang, Jie Song, Weiwei Chen, Ke Liu, Bin Liu, Peiyang Luo, Xiaohui Sun, Zhixing He, Yingying Mao, Ding Ye

**Affiliations:** ^1^Department of Epidemiology, School of Public Health, Zhejiang Chinese Medical University, Hangzhou, China; ^2^Department of the Fourth Clinical Medicine, Zhejiang Chinese Medical University, Hangzhou, China; ^3^Department of Basic Medical Sciences, Zhejiang Chinese Medical University, Hangzhou, China

**Keywords:** irritable bowel syndrome, Mendelian randomization, modifiable factors, genetics, single-nucleotide polymorphisms

## Abstract

**Background:**

Modifiable factors were found to be associated with the risk of irritable bowel syndrome (IBS) in observational studies, but whether these associations are causal is uncertain. We conducted a Mendelian randomization (MR) study to systematically explore the causal associations of modifiable factors with IBS.

**Methods:**

Summary-level statistical data for IBS was obtained from a genome-wide association study (GWAS) meta-analysis of UK Biobank (40,548 cases and 293,220 controls) and the international collaborative Bellygenes initiative (12,852 cases and 139,981 controls). Genetic instruments associated with the exposures at the genome-wide significance (*p* < 5 × 10^−8^) level were selected from previous GWASs. Mendelian randomization was performed using inverse-variance weighted (IVW) method, supplemented with several sensitivity analyses to evaluate potentially causal relationships between identified contributing factors and IBS. Furthermore, we applied another database from FinnGen (8,116 IBS cases and 276,683 controls) to testify the reliability of the significant associations.

**Results:**

Seven convincing modifiable factors were significantly associated with IBS after correction for multiple testing. Genetically predicted smoking initiation (OR = 1.12, 95% CI = 1.06–1.18, *p* = 1.03 × 10^−4^), alcohol consumption (OR = 0.47, 95% CI = 0.34–0.64, *p* = 3.49 × 10^−6^), sedentary behavior (OR = 1.17, 95% CI = 1.07–1.28, *p* = 4.02 × 10^−4^), chronotype (OR = 0.92, 95% CI = 0.88–0.96, *p* = 4.42 × 10^−4^), insomnia (OR = 1.19, 95% CI = 1.15–1.24, *p* = 7.59 × 10^−19^), education (OR = 0.80, 95% CI = 0.74–0.88, *p* = 5.34 × 10^−7^), and visceral adiposity (OR = 1.15, 95% CI = 1.06–1.24, *p* = 7.96 × 10^−4^). We additionally identified several suggestive factors, including serum magnesium, serum phosphorus, physical activity, lifetime smoking, intelligence, lean body mass, and body mass index (BMI). After pooling the effect estimates from FinnGen, the associations remained significant except for chronotype.

**Conclusion:**

This MR analysis verified several modifiable risk factors for IBS, thus prevention strategies for IBS should be considered from multiple perspectives on these risk factors.

## Introduction

Irritable bowel syndrome (IBS) is a functional gastrointestinal disorder that is estimated to affect around 1 in 10 people globally (1), while community prevalence appears to vary widely across different countries, partially due to the heterogeneity of diagnostic criteria and methodology (2). To be noted, the discrepancy also suggests a role for lifestyle factors in disease development (3).

Previous observational studies have summarized a large number of potential risk factors of IBS, involving micro-nutritional, behavioral, and obesity-related factors. For example, Barbalho et al. (4) have reported that vitamin D was implicated in the pathology of IBS. Additionally, there was consistent evidence to link subjectively reported poor sleep with IBS based on a systematic review (5). Besides, a well-conducted twin study from Norwegian found that low birth weight was associated with increased risk of IBS, as well as earlier onset of symptoms (6). However, whether causal associations between the potential risk factors and the development of IBS exist remains unanswered, because these findings could be prone to reverse causation and confounding factors.

The Mendelian randomization (MR) approach, using genetic variants as instrumental variables (IV) for exposure, can overcome the limitations of conventional observational studies (7). Since the genotype is determined at conception, which cannot be influenced by any stage of the disease process, therefore MR avoids the bias of reverse causality (8). On the other hand, alleles are randomly assigned when passed from parents to offspring during meiosis, so the genotype distribution in the population is unrelated to the presence of confounders, such as environmental exposure, socioeconomic status, and behavior.

In the current study, we used MR design to assess the possible causal relationship of modifiable factors (micro-nutritional, behavioral, and obesity-related factors) through a comprehensive search of relevant studies with risk of IBS.

## Methods

### Study design and data source

**Figure 1:**
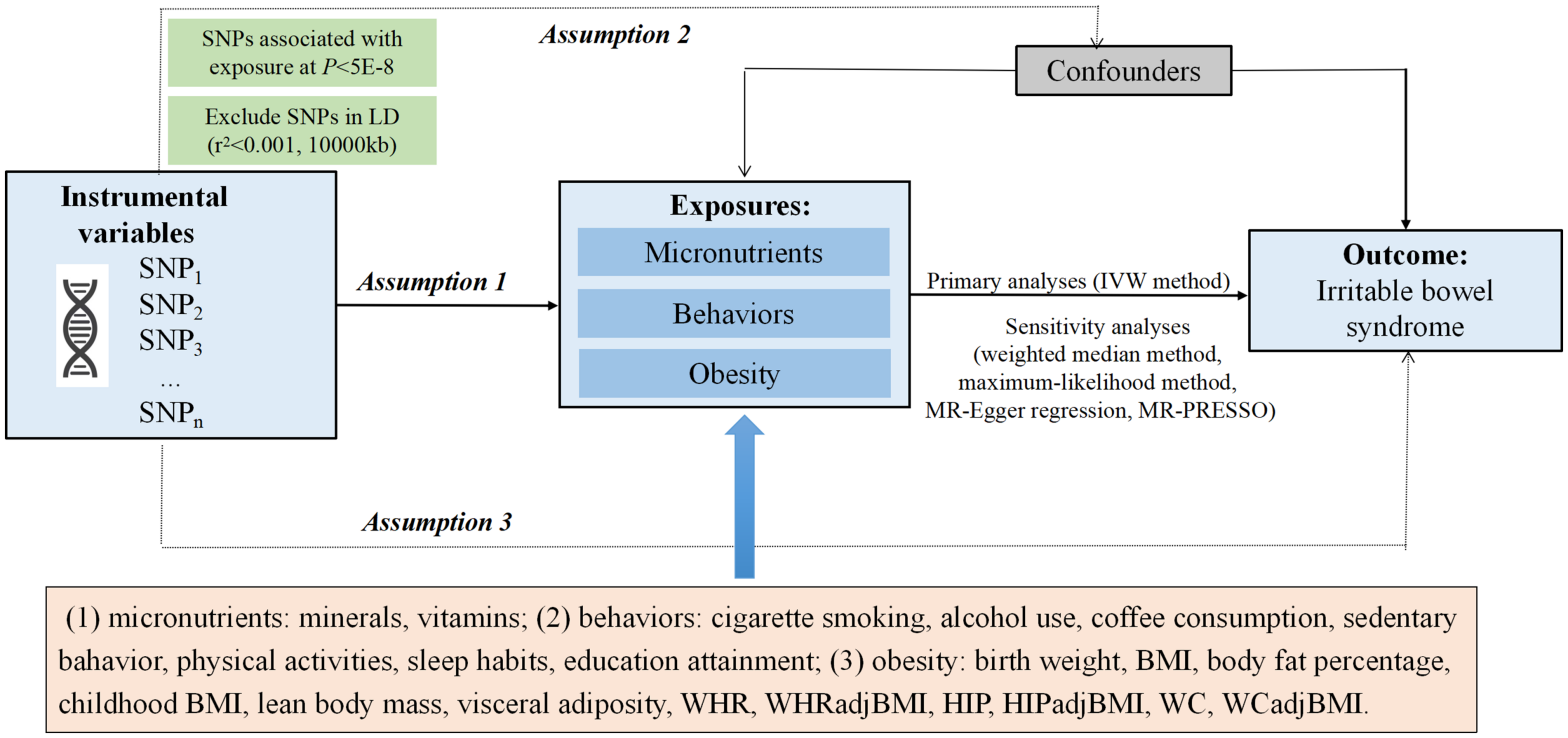
Overview of the Mendelian randomization (MR) design. Assumption 1 indicates that the genetic variants selected as instrumental variables (IVs) should be strongly associated with the exposure, assumption 2 indicates that the IVs should not be associated with confounders, and assumption 3 indicates that the IVs should affect the risk of the outcome only through the risk factor, not via alternative pathways. IVW, inverse-variance weighted; BMI, body mass index; WHR, waist-to-hip ratio; WHRadjBMI, waist-to-hip ratio adjusted for BMI; HIP, hip circumference; WC, waist circumference.

To identify possible modifiable factors for IBS, we searched relevant system reviews, meta-analysis, and observational epidemiological studies in the PubMed database from inception up to March 20, 2022. We also conducted a review of published MR studies and excluded the traits reported until March 21, 2022, including copper, zinc, gluten-free diet, asthma (9–12). Finally, we focused on studies that have reported the association between potentially modifiable factors with available IV information and IBS risk, totaling 46 factors in our MR study (Supplementary Table S1). The summary of the study design is presented in Figure 1. The genetic instruments associated with putative risk factors were identified from the public genome-wide association studies (GWASs) of European ancestry (Supplementary Table S2).

The first summary-level data of IBS GWAS was obtained from a GWAS meta-analysis of UK Biobank (40,548 cases and 293,220 controls) and the international collaborative Bellygenes initiative (12,852 cases and 139,981 controls) (13). The diagnostic criteria for the GWAS was based on Rome III symptom data, self-reported medical IBS diagnosis or electronic medical records. Moreover, we used another dataset from FinnGen^1^ involved 8,116 IBS cases and 276,683 controls, to validate the significant associations with risk of IBS and combined the results to strengthen the robustness. The diagnostic criteria for GWAS from FinnGen was based on codes of the International Classification of Diseases 9th Revision (ICD-9) and ICD-10. No ethical approval was required since we used publicly available summary data. The analytical process was in line with the STROBE-MR guidelines (14).

### Selection of genetic instruments

We selected independent single-nucleotide polymorphisms (SNPs) achieving the genome-wide significance threshold from corresponding GWASs as genetic instruments. SNPs in high linkage disequilibrium (LD) were pruned using a threshold of *r*^2^ < 0.001, distance = 10,000 kb. The retained SNPs with the lowest *p*-values for exposure were utilized as IVs. Detailed information of the instrumental SNPs is listed in Supplementary Table S3.

To be noted, several IVs came from UK Biobank and most cases (40,548 in 53,400, up to 76%) in the GWAS by Eijsbouts et al. (13) were also from UK biobank. Sample overlap may be biased in the direction of the observational association between the exposure and outcome (15). Therefore, we calculated the bias of these overlapped sample using a web tool.^2^ The estimated bias was negligible (< 0.1%), suggesting sample overlap may not substantially bias estimates (Supplementary Table S4).

### Statistical analysis

*F*-statistics and *R*^2^ were used to evaluate the strength of the genetic instruments, with an *F*-statistics >10 considered adequate to avoid weak-instrument bias (16), and *R*^2^ standing for the percentage of variation in exposures explained by the instruments (17). The *R*^2^ and *F*-statistics were calculated using the following formular:


$$\begin{array}{l} F=R^{2}\left(n-k-1\right)/k\left(1-R^{2}\right)\\ R^{2}=2\beta^{2}\times MAF\times \left(1-MAF\right)/2\beta^{2}\times MAF\times \\ \left(1-MAF\right)+\left(se{\left(\beta \right)}^{2}\times 2n\times MAF\times \left(1-MAF\right)\right) \end{array}$$

Where k is the number of variants, n is the sample size, MAF is the minor allele frequency. In addition, we undertook power calculation using a webtool^3^ (18).

In the MR analyses, we used inverse variance weighted (IVW) method for primary analysis, which provides an unbiased estimate in the absence of horizontal pleiotropy or when horizontal pleiotropy is balanced (19). Cochran’s Q test was used to evaluate the heterogeneity of the IVs. The random-effects model was applied if the Q statistic at the *p* < 0.05 level, otherwise, the fixed-effects model was utilized (20, 21).

Considering the IVW method may be invalid in the presence of pleiotropic IVs, we also performed sensitivity analysis using the weighted median and maximum likelihood methods. Weighted median method can provide valid causal estimates if more than 50% of the IVs are valid (22). Maximum-likelihood method produces estimates for the probability distribution parameters by maximizing the likelihood function with low standard error (23). Besides, the intercept obtained from the MR-Egger regression was used to evaluate the horizontal pleiotropy, with a *p* < 0.05 suggesting the presence of pleiotropy (16). Moreover, we applied MR pleiotropy residual sum and outlier (MR-PRESSO) method to detect and correct for potential outliers (24). The minimum numbers of instrumental SNPs allowed for the MR analysis were two, three, two, three and four for IVW, weighted median, maximum likelihood, MR-Egger and MR-PRESSO methods, respectively. For exposure with only one instrumental SNP, we used Wald ratio to estimate the association with risk of IBS.

All analyses were conducted using R (version 4.1.2) with “Mendelian Randomization” (25) and “MR-PRESSO” (24) packages. Results were reported as OR with corresponding 95% CIs. An observed *p*-value <1.09 × 10^−3^ (0.05 divided by 46 risk factors) was considered statistically significant based on the Bonferroni correction for multiple comparisons. *p*-values between 1.09 × 10^−3^ and 0.05 were considered as suggestive associations. Subsequently, we validated the significant findings using another independent database from FinnGen. Finally, we combined the estimates for each influencing factor from the two databases using the random-effects meta-analysis method, with *p* value <0.05 as the threshold for statistical significance.

## Results

The number of SNPs ranged from 1 to 425, and the explained variances varied from 0.1 to 6.6%. The F-statistics range from 10.94 to 432.49, suggesting that all SNPs had sufficient validity. Further, we performed power calculations based on the sample size of the IBS datasets. The detailed variances, F-statistics and power are displayed in Supplementary Table S2.

**Figure 2:**
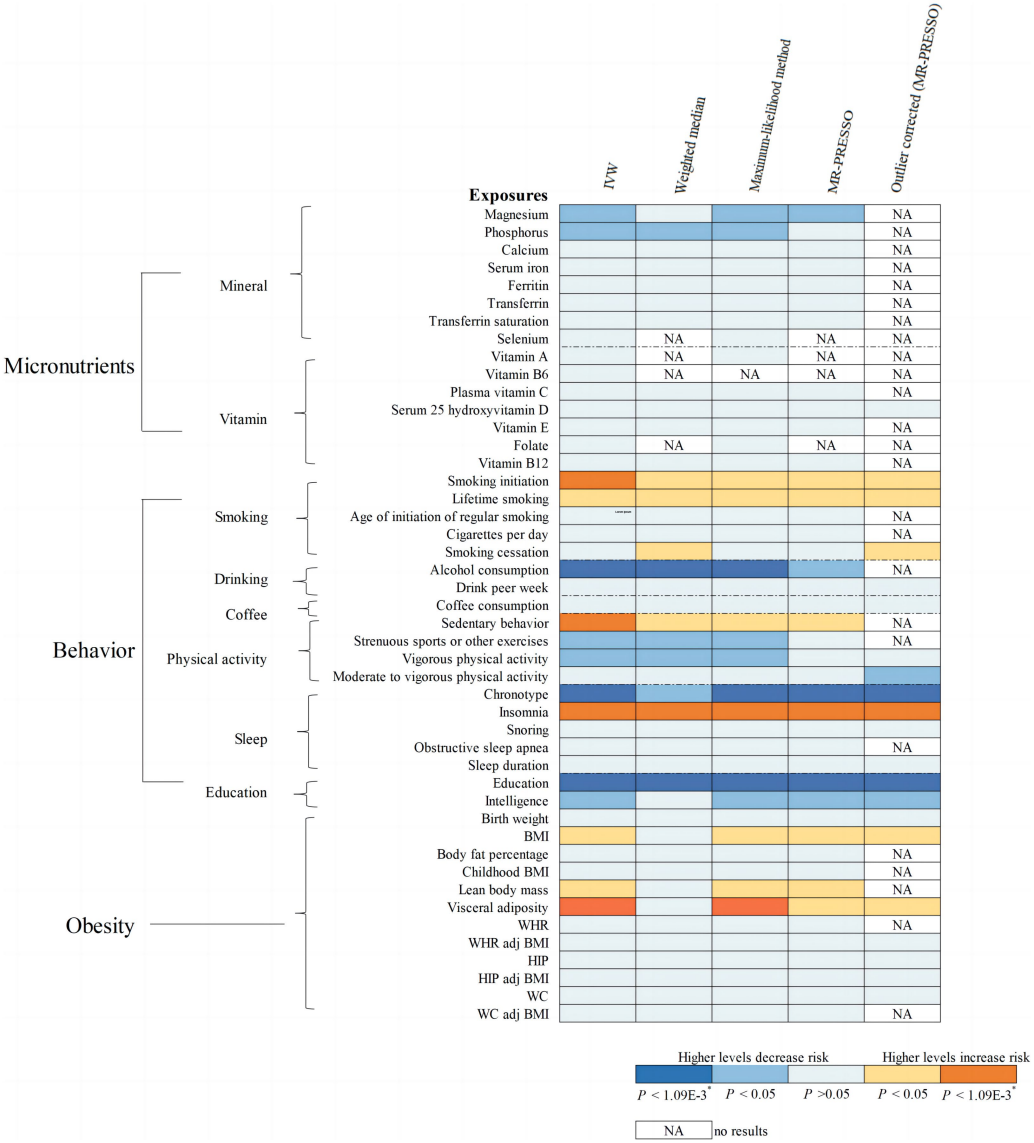
MR associations of 46 potential modifiable factors with irritable bowel syndrome (IBS). The results shown are derived from the primary method and sensitivity methods.

**Figure 3:**
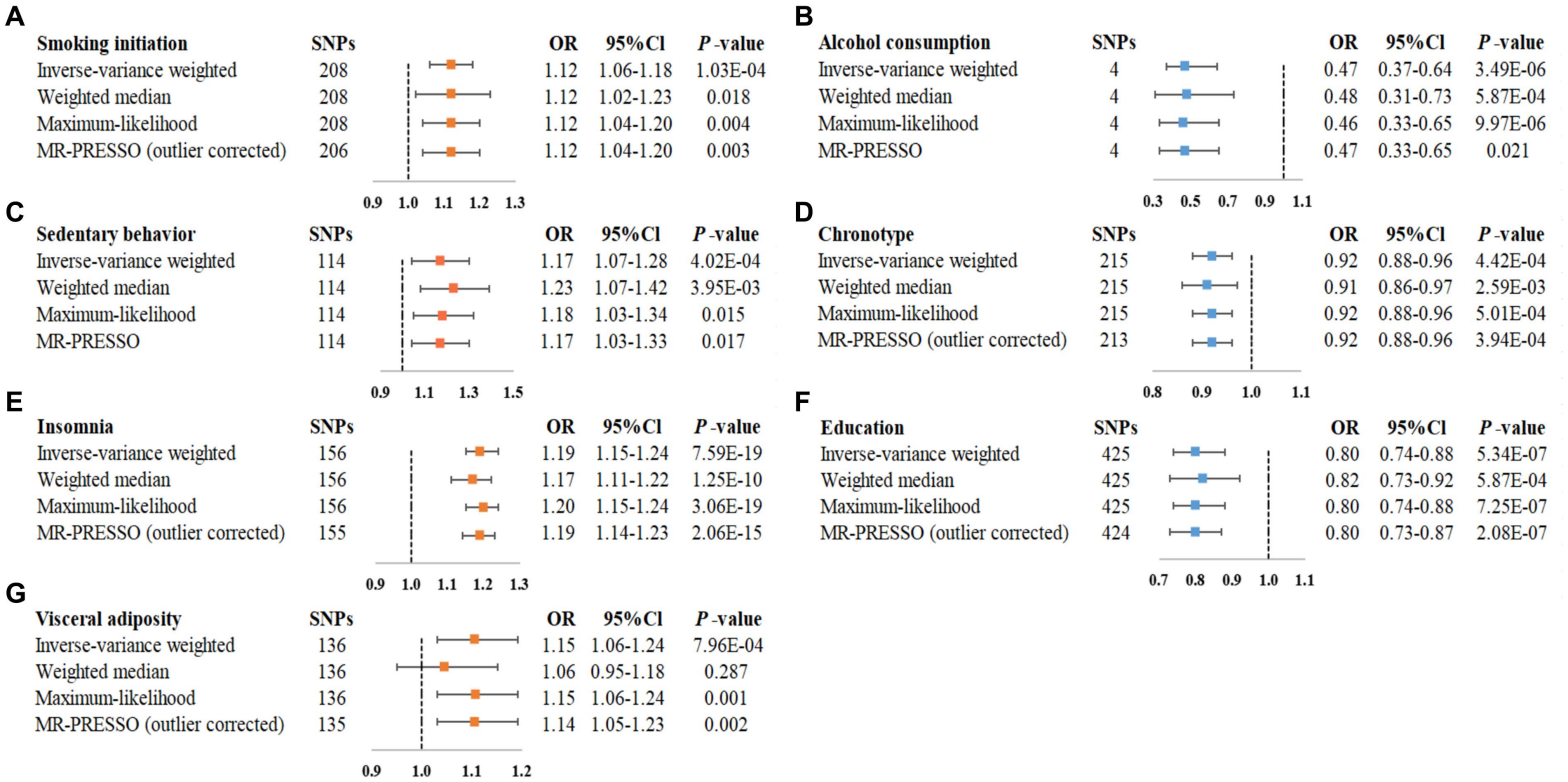
Statistically significant associations of genetically predicted modifiable factors and IBS in main and sensitivity analyses. **(A)** smoking initiation-IBS, **(B)** alcohol consumption-IBS, **(C)** sedentary behavior-IBS, **(D)** chronotype-IBS, **(E)** insomnia-IBS, **(F)** education-IBS, **(G)** visceral adiposity-IBS.

Overall, the MR associations of 46 modifiable factors with IBS derived from the IVW method are shown in Figure 2. Seven convincing factors were significantly associated with IBS risk after correction for multiple testing, and eight suggestive factors was potentially associated with the risk of IBS, with sensitivity analyses giving similar results (Figure 3; Supplementary Table S5). Specifically, genetically predicted smoking initiation, sedentary behavior, insomnia, visceral adiposity were statistically linked to higher IBS risk, while lifetime smoking, lean body mass, BMI were suggestively associated with increasing risk of IBS. Conversely, genetically predicted alcohol consumption, chronotype, education were statistically associated with reduced IBS risk, while serum magnesium and phosphate, intelligence, physical activity were suggestively associated with decreasing risk of IBS. Of the 15 exposures, a total of 14 modifiable factors have been confirmed in the meta-analysis (Supplementary Figure S1). MR analysis revealed non-significant association of some factors on the risk of IBS (Supplementary Table S6). Detailed information about heterogeneity between the SNPs used as instrumental variables for these associations is listed in Supplementary Tables S5, S6.

### Micronutrients

Regarding the 8 minerals and 7 vitamins investigated, a suggestive association was observed that genetically predicted serum magnesium (Mg) concentration was associated with a 67% (95% CI =19–87%) lower risk of IBS (Figure 2). We also noted a suggestive association between per 1-SD increment raised serum phosphorus concentrations and 28% decreased risk of IBS (95% CI = 10–43%; Figure 2). Sensitivity analysis provided similar results for Mg and phosphors (Supplementary Figure S2). These associations of serum Mg and phosphorus with the risk of IBS remained consistent in the combined analysis, with ORs of 0.37 (95% CI = 0.16–0.87) for serum Mg and 0.79 (95% CI = 0.64–0.98) for serum phosphorus (Supplementary Figure S1). However, our study showed no association of the other minerals or vitamins with the risk of IBS (Supplementary Table S6).

### Behavior

Genetically predicted smoking initiation was positively correlated with an increased risk of IBS (OR = 1.12, 95% CI = 1.06–1.18; Figure 2). Horizontal pleiotropy was not observed by MR-Egger (*P* for intercept = 0.621), and the other MR sensitivity approaches provided similar risk estimates (Figure 3). Two outliers were detected by the MR-PRESSO test, and the direction and magnitude of the association did not change (Figure 3). Also, genetically predicted lifetime smoking showed a suggestive association with the risk of IBS (OR = 1.17, 95% CI = 1.01–1.35; Figure 2). Results were consistent in sensitivity analyses (Supplementary Figure S2). After combining the associations from two datasets, the ORs for smoking initiation and lifetime smoking were 1.12 (95% CI = 1.06–1.18, Supplementary Figure S1) and 1.18 (95% CI = 1.04–1.33, Supplementary Figure S1), respectively.

As depicted in Figure 2, our MR analyses revealed a statistically significant association between genetically predicted alcohol consumption and lower risk of IBS (OR = 0.47, 95% CI = 0.34–0.64). Sensitivity analyses demonstrated consistent directions and similar effect estimates to IVW, with no evidence of unbalanced pleiotropy (*P* for intercept from MR-Egger = 0.198, Figure 3). The observed association between alcohol consumption and IBS was consistent in the meta-analysis of two databases (OR = 0.45, 95% CI = 0.34–0.61, Supplementary Figure S1).

Regarding the physical activity factors investigated, under the IVW estimation model, we noted a statistically significant association between genetically predicted sedentary behavior and a higher risk of IBS (OR = 1.17, 95% CI = 1.07–1.28; Figure 2). The association remained stable in sensitivity analyses (Figure 3), with no evidence of unbalanced pleiotropy (*P* for intercept from MR-Egger = 0.069). On the contrary, genetically predicted strenuous sports or other exercises (SSOE) (OR = 0.88, 95% CI = 0.78–0.99; Figure 2) as well as vigorous physical activity (VPA) (OR = 0.86, 95% CI = 0.75–0.99; Figure 2) were inversely associated with the risk of IBS, and sensitivity analysis yielded consistent results (Supplementary Figure S2). The results from the meta-analysis of two databases confirmed the findings of sedentary behavior (OR = 1.26, 95% CI = 1.05–1.50, Supplementary Figure S1), strenuous sports or other exercises (OR = 0.88, 95% CI = 0.78–0.99, Supplementary Figure S1), and vigorous physical activity (OR = 0.86, 95% CI = 0.75–0.99, Supplementary Figure S1).

As for the sleep-related traits, we observed that genetically predicted insomnia was significantly associated with increased risk of IBS by IVW method (OR = 1.19, 95% CI = 1.15–1.24, Figure 2), and sensitivity analyses yielded consistent results (Figure 3). MR-Egger regression indicated no potential pleiotropic bias for the IVs (*P* for intercept = 0.185). In addition, genetically predicted chronotype was inversely associated with the risk of IBS (OR = 0.92, 95% CI = 0.88–0.96; Figure 2). Results of risk estimates were consistent in sensitivity analyses (Figure 3). MR-Egger intercept test indicated no evidence of directional pleiotropy (*P* for intercept = 0.420). By using the MR-PRESSO test to exclude possible outliers, the effect estimates of the association between chronotype and risk of IBS did not change markedly (Figure 3). The association of insomnia with risk of IBS has been confirmed in the meta-analysis (OR = 1.19, 95% CI = 1.15–1.24, Supplementary Figure S1), but the result of chronotype did not reach significance (OR = 1.00, 95% CI = 0.85–1.17, Supplementary Figure S1).

Genetically predicted higher educational attainment was associated with significantly lower risk of IBS (OR = 0.80 per SD increase in years of education completed, 95% Cl = 0.74–0.88; Figure 2). The association was confirmed in the sensitivity analyses (Figure 3). In addition, genetically intelligence was suggestively correlated with a reduced risk of IBS (OR = 0.91, 95% CI = 0.84–0.99) (Supplementary Figure S2). MR-PRESSO identified several outliers, but a similar estimate was observed after the removal (Supplementary Figure S2). The combined effects of educational attainment and intelligence on risk of IBS were 0.76 (95% CI = 0.66–0.88, Supplementary Figure S1) and 0.91 (95% CI = 0.84–0.97, Supplementary Figure S1), respectively.

### Obesity

IVW analysis showed the association between genetically determined visceral adiposity and IBS was statistically significant (OR = 1.15, 95% CI = 1.06–1.24, Figure 2). Similar results were obtained in sensitivity analyses (Figure 3). Additionally, after the removal of some outliers identified by the MR-PRESSO test, the magnitude of the effect and their precision remained similar (Figure 3). Except for visceral adiposity, the MR analysis also revealed an adverse effect of genetically predicted lean body mass (OR = 1.07, 95% CI = 1.02–1.12, Figure 2) or BMI (OR = 1.08, 95% CI = 1.03–1.15, Figure 2) on IBS. Consistent results were obtained through sensitivity analyses (Supplementary Figure S2). There were consistent associations for BMI (OR = 1.08, 95% CI = 1.03–1.14), lean body mass (OR = 1.06, 95% CI = 1.01–1.10), and visceral adiposity (OR = 1.15, 95% CI = 1.07–1.23) in the pooled analysis (Supplementary Figure S1).

## Discussion

In summary, in this study, we applied MR approach to comprehensively investigate potential risk factors that might have causal effects on the development of IBS. Specifically, our study noted that genetically predicted smoking initiation, alcohol consumption, chronotype, insomnia, educational attainment, sedentary behavior and visceral adiposity are causally associated with risk of IBS. There was also suggestive evidence for possible association of magnesium, phosphorus, lifetime smoking, physical activity, intelligence, lean body mass, and BMI with the risk of IBS.

Previous studies have shown that smoking might be a potential risk factor for IBS, though the association was controversial. For example, a systematic review of 55 articles reported that the association between smoking and IBS cannot be confirmed (26). While a meta-analysis based on three population-based studies in Sweden reported that smokers have higher risk of developing IBS-diarrhea (OR = 2.40, 95% CI 1.12–5.16) than non-smokers, which is in line with our study (27). Cigarette smoking has been associated with detrimental effects on both visceral and peripheral hypersensitivity and gastrointestinal motility (28). On the other hand, smoking may pose an adverse effect on intestine through microbiome (29). These changes in microbiota may also be of importance for IBS. To be specific, incorporating smoking cessation programs into IBS preventive strategies is essential to mitigate the adverse effects of smoking on gut health.

It is well known that alcohol can interfere with many functions of the gut including damage to the mucosa of the intestine, increased intestinal membrane permeability and visceral sensitivity, disturbed motility of the gut, composition and activity of the microbiota (30). A population-based study of 2,648 participants reported an inverse association for drinking frequency of 2–3 times a week (OR = 0.352, 95% CI = 0.123–1.006) and drinking 3–4 standard glasses per occasion (OR = 0.716, 95% CI = 0.522–0.982) with IBS (31). Also, in a population-based study of middle-age and elder subjects showed a tendency toward less symptoms with a moderate alcohol intake (28). Similarly, a case–control study of women aged 18–48 years reported weaker symptoms with moderate and light alcohol intake, whereas aggravated symptoms after high intake of alcohol (32). Therefore, the relationship between alcohol consumption and risk of IBS may be nonlinear, while MR analysis was not able to measure potentially nonlinear relationship, so further studies are warranted. Comprehensive prevention plans for IBS should include measures to educate individuals on moderate alcohol consumption, avoidance of excessive drinking, and provision of support for alcohol cessation, ensuring personal health and welfare.

More generally, physical activity has a positive impact on gastrointestinal disorders. Consistent with our study, a cross-sectional study consisting of 4,763 Iranian adults suggested that the time spent in sedentary behavior per day is positively associated with a higher risk of IBS (OR = 1.27, 95% CI = 1.08–1.49), especially among women and individuals of normal weight (33). Previous studies have reported that spending more time in moderate physical activity is associated with improvement of symptoms (31, 34). The mechanisms behind the association have not been fully understood. One of the possible explanation is that physical activity may have protective effects on the gastrointestinal tract such as decreased gastrointestinal blood flow, increased gastrointestinal motility, increased mechanical bouncing and neuro-immuno-endocrine alterations (35). Overall, further studies are warranted to shed light on this association. Engaging in regular physical activity is linked to enhanced gut motility and lower stress levels, both of which can aid in the prevention of IBS.

Sleep disorders are symptoms frequently reported by patients with IBS, which may be associated with greater gastrointestinal symptom severity (36, 37). Recently, a meta-analysis including 11 studies from seven countries reported that shift work (OR = 2.27, 95% CI = 1.674–3.067) and poor sleep quality (OR = 4.27, 95% CI = 2.79–6.53) are significant risk factors for medical staff suffering from IBS (38). However, the observed association might be biased by reverse causation. Fortunately, a systematic review of 5 *p*rospective studies concluded that sleep disorders are risk factors for IBS (39). Our study provided more robust evidence for the causal association of insomnia and chronotype with risk of IBS. The underlying mechanism linking sleep disturbance and IBS remain unclear. It was proposed that disruption of circadian physiology, due to shift work or sleep disturbance, may lead to increased permeability of the intestinal, alteration and dysregulation of immune and inflammatory responses (40, 41). It is recommended to address and ameliorate sleep disturbances as a strategy to prevent IBS.

Epidemiological findings for the association between educational attainment and risk of IBS were inconsistent. Several studies showed that low educated is associated with a higher prevalence of IBS (42), while Faresjö et al. (43) found no significant differences between IBS patients and healthy controls in the education level by case–control design. These conflicting results might be attributed to the study design, definitions differences, cultural differences, environmental factors, and small sample size. MR methods are more robust to provide evidence for the protective effects of educational attainment on the risk of IBS. This effect can be explained that higher levels of educational attainment are associated with health behaviors (e.g., physical activity, diet, and smoking), more cognitively-complex occupations, and better access to health care, all of which may play a role in decreasing risk of various diseases (44). Additionally, education plays a pivotal role in raising awareness about health issues, encouraging proactive measures for prevention and treatment of IBS. Investing in education is crucial in combating the prevalence of IBS and promoting overall well-being.

Obesity seems to be involved in the pathogenesis of IBS, but the previous findings were conflicting. BMI, a commonly used indicator of general obesity, has been reported to be associated with an increased risk of IBS according to a population-based study (45). However, a birth cohort study reported that increased BMI was not significantly associated with IBS (46). Given the nature of observational analysis, association observed may be due to residual or unmeasured confounding, therefore, we applied a MR approach to circumvent these limitations. The results presented here provide more convincing evidence supporting the deleterious effect of obesity on IBS, and several mechanisms may link obesity and IBS disease ranging from the disorder of intestinal motility, gut microbiota alteration, and inflammation factors. Specifically, it is possible that excess body weight increases intra-abdominal pressure and subsequently causes abnormal IBS motility mechanically (47). Second, obesity has been associated with changes in the microbiota composition, confirming the possible link with IBS (48). Moreover, visceral adipose tissue secretes a number of adipokines and cytokines leading to tissue inflammation (49). Incorporating weight control strategies into IBS preventive plans is paramount due to the significant impact of weight on gut health.

Furthermore, several suggestive risk factors were identified in the present study. Our study found that serum Mg and phosphorus concentrations decrease IBS risk by 67 and 28% per one SD increase respectively, but the association of Mg or phosphorus with the risk of IBS is inconclusive. Previous studies have shown that the diet consumed by IBS patients has low magnesium and phosphorus content (50). Roth et al. (51) reported that extraintestinal symptoms and fatigue for IBS patients were inversely associated with intakes of Mg and phosphorus. However, the exact mechanism by which Mg or phosphorus exerts its protective effect in IBS still remains unclear, and further investigation is necessary. Moreover, we found a suggestive association between genetically predicted intelligence and risk of IBS. According to a systematic review of twelve studies, there was inconclusive evidence to conclude the relationship between intelligence (including Verbal IQ, Performance IQ and experiential intelligence) and risk of IBS (52). We speculated this result may be attributed to little considerations for confounding factors. Given the tight phenotype and genetic correlation between intelligence and education level, the underlying mechanisms may be similar.

This is the first study that comprehensively assessed the causal associations between multiple exposures and risk of IBS by exploiting data from large GWASs. Many of the putative risk factors considered in this study have not previously been assessed within MR frameworks. The use of the MR design strengthened the causal inference on the exposure-IBS associations due to diminished residual confounding and reverse causality. In addition, several sensitivity analyses were performed to test the consistency of results, and reveal and correct for possible pleiotropy.

The current study has several limitations that should be considered when interpreting our findings. For MR analysis, the validity relies on the following three assumptions. The first assumption is that the genetic variants selected as IVs should be strongly associated with the modifiable risk factors. To satisfy this assumption, we selected SNPs achieving the genome-wide significant threshold, ensuring a robust association between these SNPs and the factors of interest. The second assumption is that the IVs should not be associated with any confounder which might influence both the exposures and the outcome. Given the alleles of genetic variants are randomly allocated at gamete formation, covariates are anticipated to be randomly distributed with respect to genotypes, making them generally free of confounders The third assumption is that the IVs should be associated with IBS only through the exposure, without any direct effects on the outcome. We confirmed by performing MR-Egger regression and finding no evidence of directional pleiotropy. First, we might have overlooked weak associations, especially for traits with small variance explained by SNPs. Second, we could not examine potential nonlinear relationships for risk factors using summary data. Third, our study was restricted to individuals of European ancestry, which limits the generalizability of our findings to diverse populations. Therefore, our conclusions may not be generalizable to other populations, as the participants of the included GWAS studies are Europeans. Moreover, sample overlap should be recognized between the GWAS studies of exposure and outcome, though the estimated bias was relatively low. Finally, it’s worth noting that the diagnostic criteria for IBS varied between the two GWAS studies, which could potentially impact the results.

## Conclusion

IBS is a complex disease caused by interaction of multiple factors and the exact pathogenesis has not been fully elucidated. Our study has verified the causal association between several modifiable risk factors and IBS from insights from MR analysis, suggesting the important role of the management of micronutrient concentration, education level, weight control, sleep quality, physical activity, and smoking rates in IBS prevention.

## Data availability statement

The datasets presented in this study can be found in online repositories. The names of the repository/repositories and accession number(s) can be found in the article/Supplementary material.

## Ethics statement

The study was approved by the Ethical Committee of Zhejiang Chinese Medical University on Nov 16, 2021 (No. AF-20211116-1). Because the project was mainly based on statistical analyses of publicly accessible databases and published studies, in which informed consents were obtained and ethical review were completed separately in each study, we have received ethical waiver for the research project. The studies were conducted in accordance with the local legislation and institutional requirements. Written informed consent for participation was not required from the participants or the participants' legal guardians/next of kin in accordance with the national legislation and institutional requirements.

## Author contributions

YC: Formal analysis, Methodology, Writing – original draft. HY: Formal analysis, Methodology, Visualization, Writing – original draft. JS: Formal analysis, Methodology, Visualization, Writing – original draft. WC: Methodology, Software, Validation, Writing – original draft. KL: Formal analysis, Methodology, Writing – original draft. BL: Data curation, Methodology, Writing – original draft. PL: Visualization, Writing – original draft. XS: Conceptualization, Supervision, Writing – review & editing. ZH: Investigation, Supervision, Writing – review & editing. YM: Writing – review & editing. DY: Conceptualization, Supervision, Validation, Writing – review & editing.

## Footnotes

^1^ https://www.finngen.fi/en

^2^ https://sb452.shinyapps.io/overlap/

^3^ https://shiny.cnsgenomics.com/mRnd/

## Supplementary material

SUPPLEMENTARY FIGURE S1 Associations of genetically predicted modifiable factors with risk of IBS in two datasets and the combined effects.

SUPPLEMENTARY FIGURE S2 Suggestive associations of genetically predicted modifiable factors and IBS in main and sensitivity analyses.
