# Supplementary figures and images for: Associations of modifiable factors with risk of irritable bowel syndrome
Source: Front Nutr. 2024 Jul 1;11:1362615. doi: 10.3389/fnut.2024.1362615 (PMC11246901; doi:10.3389/fnut.2024.1362615)

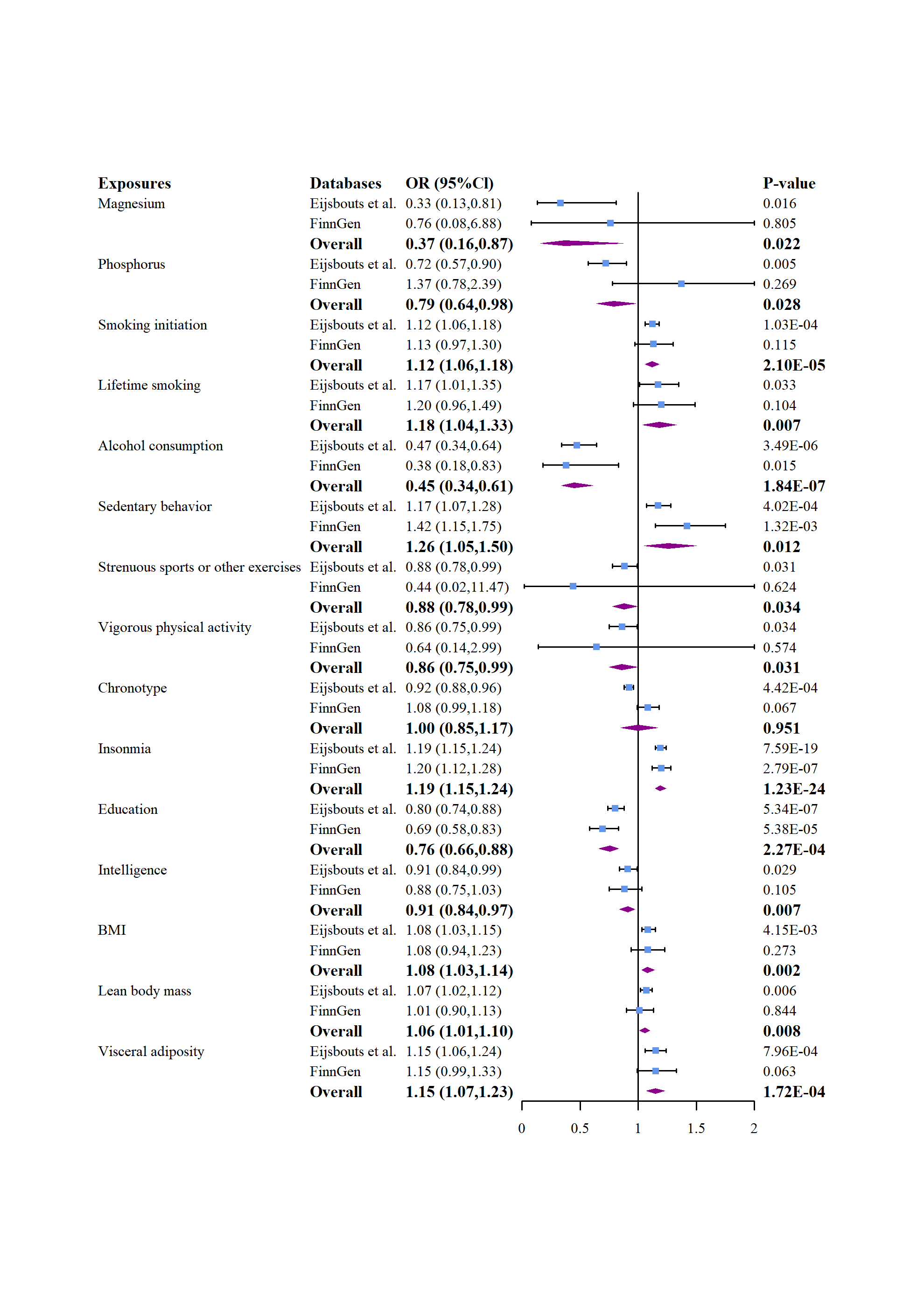

Supplement: SUPPLEMENTARY FIGURE S1 — Associations of genetically predicted modifiable factors with risk of IBS in two datasets and the combined effects. [file Image_1.TIFF]

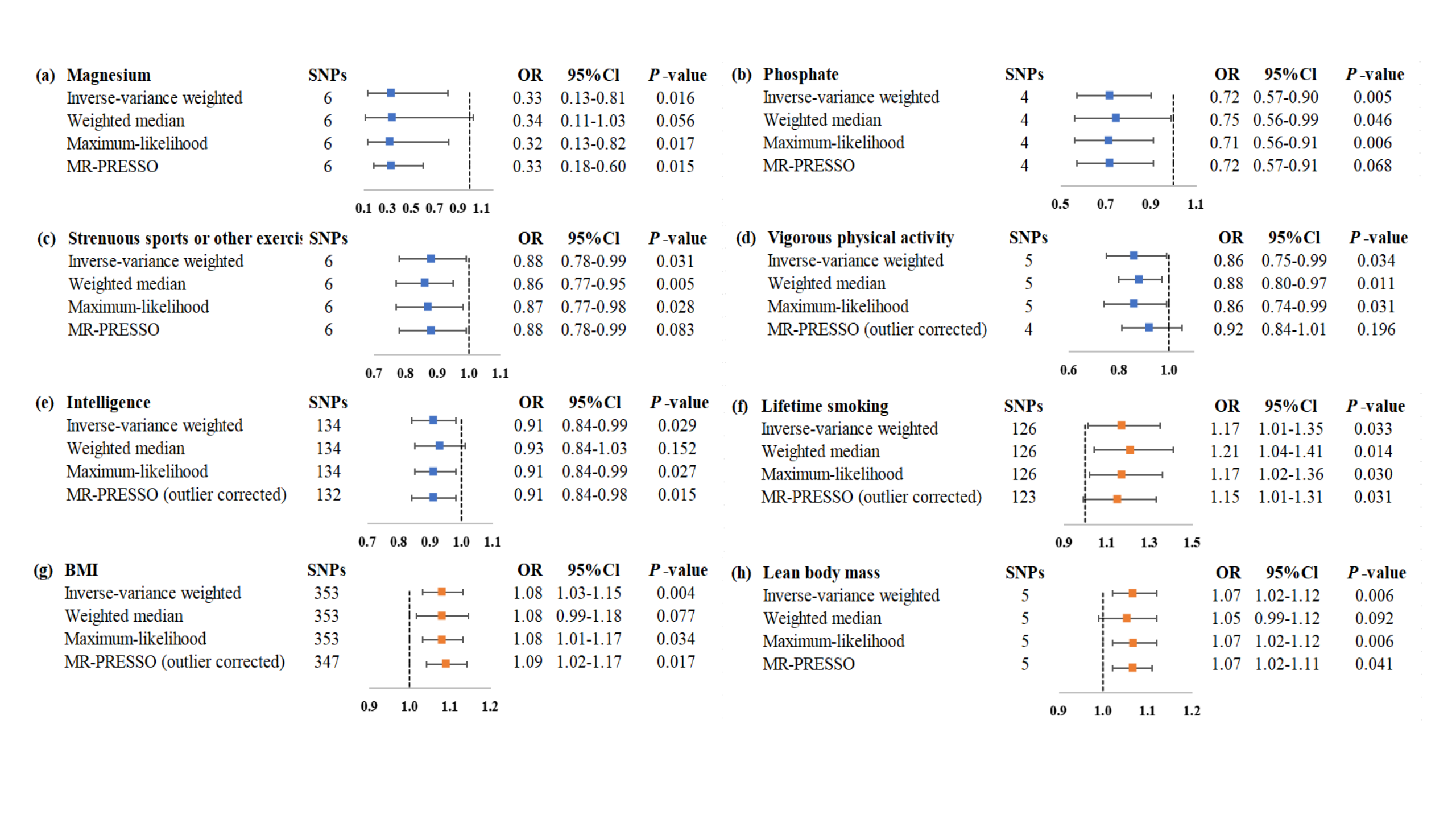

Supplement: SUPPLEMENTARY FIGURE S2 — Suggestive associations of genetically predicted modifiable factors and IBS in main and sensitivity analyses. [file Image_2.TIF]
